# Crystal structure and Hirshfeld surface analysis of aqua­(1*H*-imidazole-κ*N*^3^)[*N*-(2-oxido­benzyl­idene)threonato-κ^3^*O*,*N*,*O*′]zinc(II)

**DOI:** 10.1107/S2056989025002385

**Published:** 2025-03-25

**Authors:** Fumishi Yoshizawa, Anna Okui, Daisuke Nakane, Takashiro Akitsu

**Affiliations:** a Department of Chemistry, Faculty of Science, Tokyo University of Science, 1-3, Kagurazaka, Shinjuku-ku, Tokyo 162-8601, Japan; University of Hyogo, Japan

**Keywords:** Schiff base ligand, zinc(II) complex, amino acid, Hirshfeld surface analysis, crystal structure

## Abstract

The title complex, [Zn(C_11_H_11_NO_4_)(C_3_H_4_N_2_)(H_2_O)], which includes a tridentate ligand, was synthesized from l-threonine and salicyl­aldehyde. One water mol­ecule and one imidazole mol­ecule additionally coordinate the zinc(II) center in a distorted trigonal–bipyramidal geometry. The crystal structure features N—H⋯O and O—H⋯O hydrogen bonds. A Hirshfeld surface analysis indicates that the most important contributions to the packing are from H⋯H/H⋯H (50.7%) and O⋯H/H⋯O (25.0%) contacts.

## Abstract 2

The crystal structure of the amino acid Schiff base zinc(II) complex synthesized from salicyl­aldehyde, l-threonine and zinc(II) acetate is reported.

## 1. Chemical context

Amino acid Schiff bases have an azomethine (C=N) group synthesized by mixing primary amines and formyls, and are used as organic ligands (Katsuumi *et al.*, 2020 ▸; Hirotsu *et al.*, 2022 ▸; Gozdas *et al.*, 2024 ▸; Bowman *et al.*, 2021 ▸). According to a review on the synthesis of amino acid Schiff base–metal complexes (Akitsu *et al.*, 2022 ▸), in general, Schiff bases and their metal complexes are versatile compounds and are widely used in many research and industrial applications. For example, supra­molecular encapsulation of nanocrystalline Schiff bases in β-cyclo­dextrin (Mahato *et al.*, 2022 ▸), photoreaction with titanium dioxide (Takeshita & Akitsu, 2015 ▸), photocatalytic reduction of hexa­valent chromium (Nakagame *et al.*, 2019 ▸; Miyagawa *et al.*, 2020 ▸), Schiff base ligand–SPCE (screen-printed carbon electrode) sensors (Bressi *et al.*, 2022 ▸), and flexible ruthenium(II) Schiff base complexes, which have been shown to play a key role in drug activity upon photoirradiation (Gillard *et al.*, 2020 ▸).

Furthermore, Schiff base complexes are considered an important class of organic compounds with a wide range of biological properties, including free-radical-scavenging activity, anti­bacterial activity, and anti­tumor activity (Kumar, 2022 ▸). In our laboratory, we synthesized novel mono-chlorinated Schiff base copper(II) complexes and tested their anti­bacterial activity against Gram-positive and Gram-negative bacteria. The most active compounds were then tested for anti­oxidant activity, and it was found that *E. coli* absorbed these compounds with very high affinity (Otani *et al.*, 2022 ▸). We are also conducting research using microfluidic devices to efficiently synthesize amino acid Schiff base copper(II) complexes (Kobayashi *et al.*, 2023 ▸), and synthesis of amino acid Schiff base copper(II) complexes containing azo­benzene moiety (Kaneda *et al.*, 2024 ▸). Our goal is to evaluate the SOD activity of artificial metalloproteins made by conjugating these Schiff base copper(II) complexes with proteins such as lysozyme (Furuya *et al.*, 2023 ▸; Nakane *et al.*, 2024 ▸).

Therefore, we have been studying the bioactivity of Schiff base complexes derived from amino acids and decided to synthesize a zinc complex of this ligand to compare its bioactivity with that of the copper complex. In this report, we describe the crystal structure and inter­molecular inter­actions of the zinc(II) complex, coordinated with imidazole as a model for histidine residues in proteins.
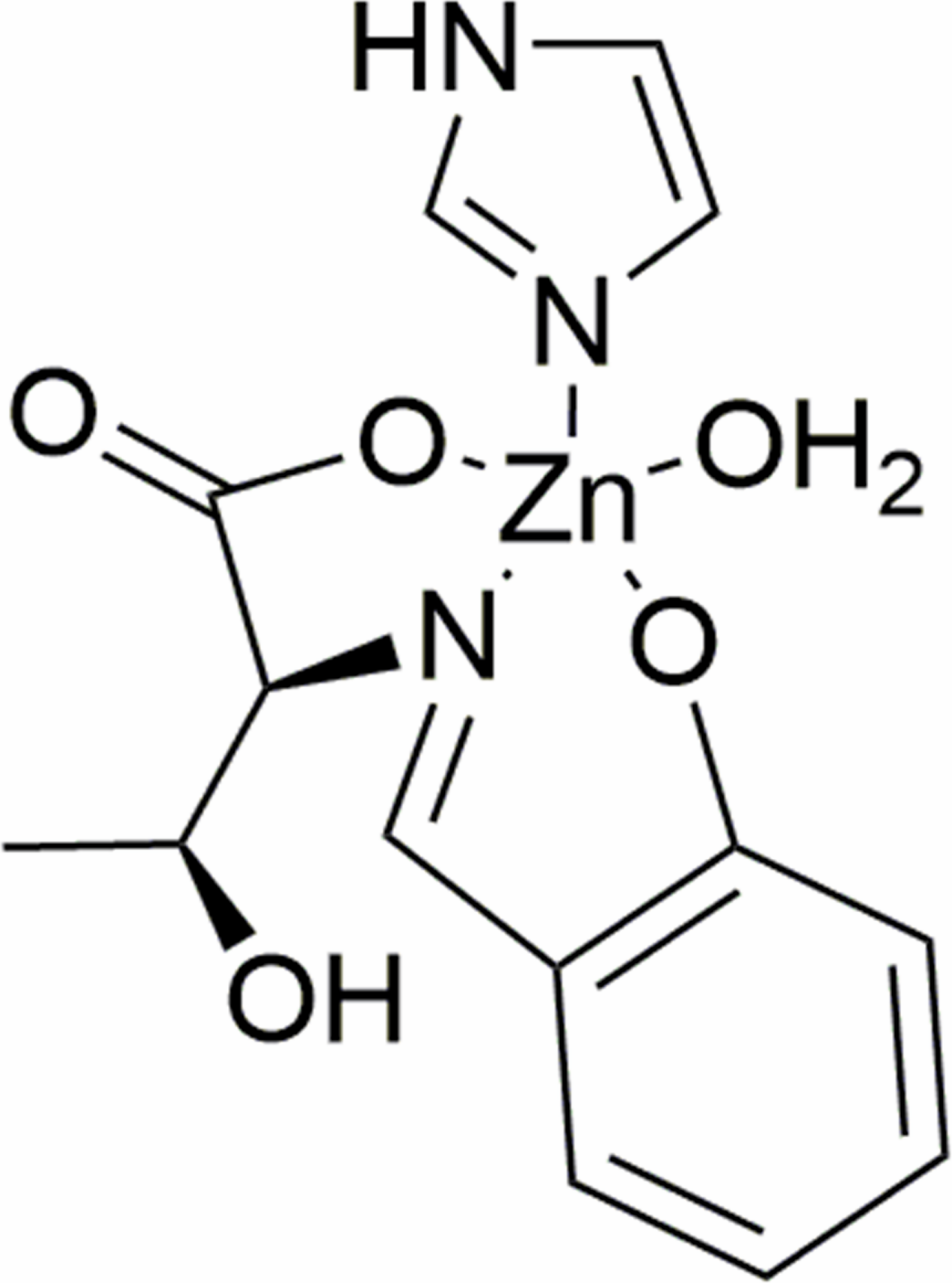


## 2. Structural commentary

**Figure 1:**
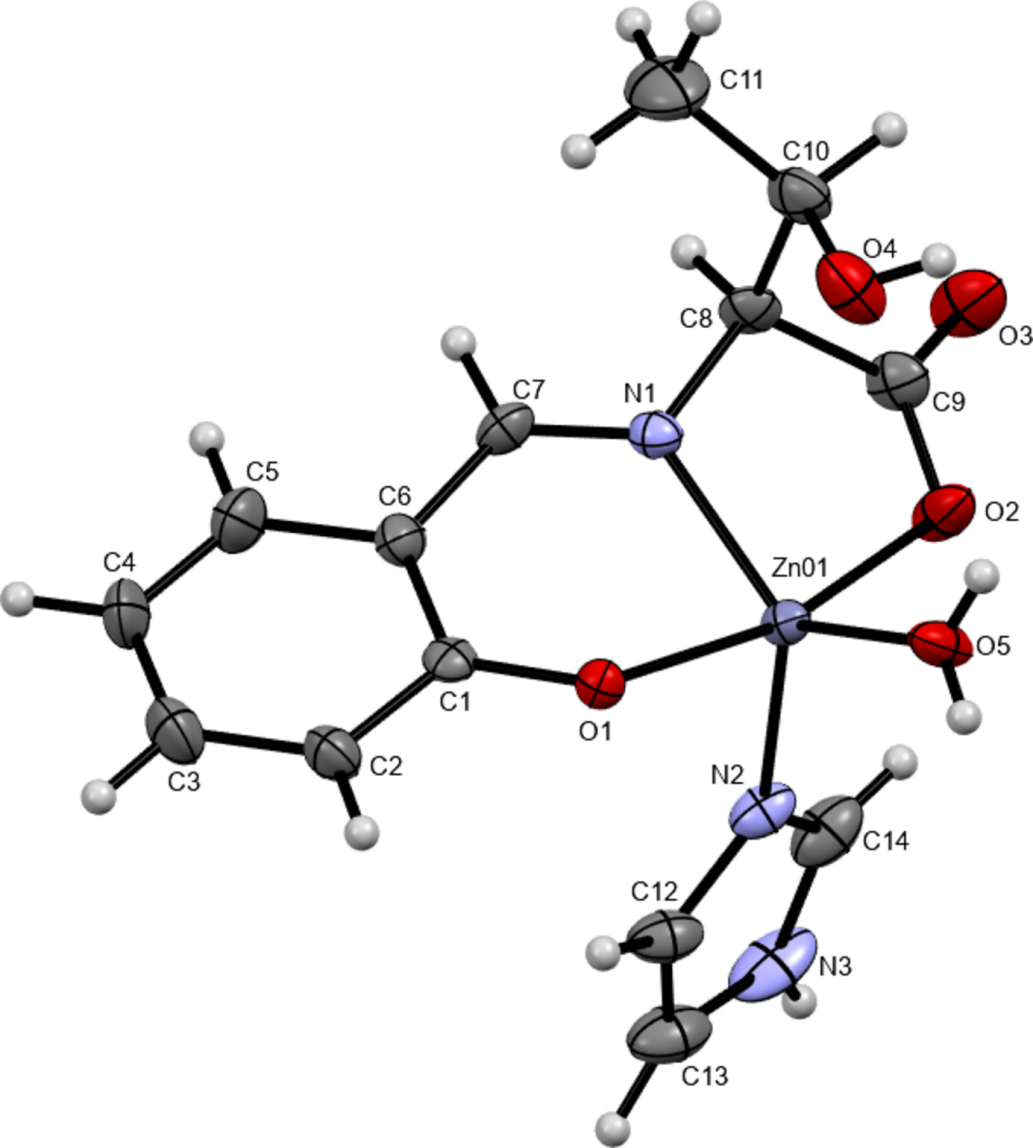
The mol­ecular structure of the title compound with ellipsoids drawn at the 50% probability level.

The mol­ecular structure of the title compound consists of one imidazole mol­ecule, one water mol­ecule and a tridentate ligand, which is synthesized from l-threonine and salicyl­aldehyde, coordinating to a zinc(II) center in distorted trigonal–bipyramidal geometry (Fig. 1 ▸). The two largest coordination angles O1—Zn01—O2 and N1—Zn01—O5 are 166.60 (11), 130.65 (13)°, and the τ value derived from them, which is five-coordinated geometry index, is 0.599 (Addison *et al.*, 1984 ▸). The C7—N1 distance is 1.276 (5) Å, which is close to a typical C=N double-bond length for an imine (Katsuumi *et al.*, 2020 ▸). The Zn01—O1, Zn01—O2 and Zn01—O5 coordination lengths are 2.061 (2), 2.117 (3) and 1.996 (3) Å, respectively, close to a typical Zn—O bond length (Noor *et al.*, 2021 ▸). The Zn01—N1 and Zn1—N2 bonds of 2.038 (3) and 2.015 (3) Å corresponds to a typical Zn—N bond length (Noor *et al.*, 2021 ▸). These five atoms coordinating to Zn1 have similar bond distances.

## 3. Supra­molecular features

**Figure 2:**
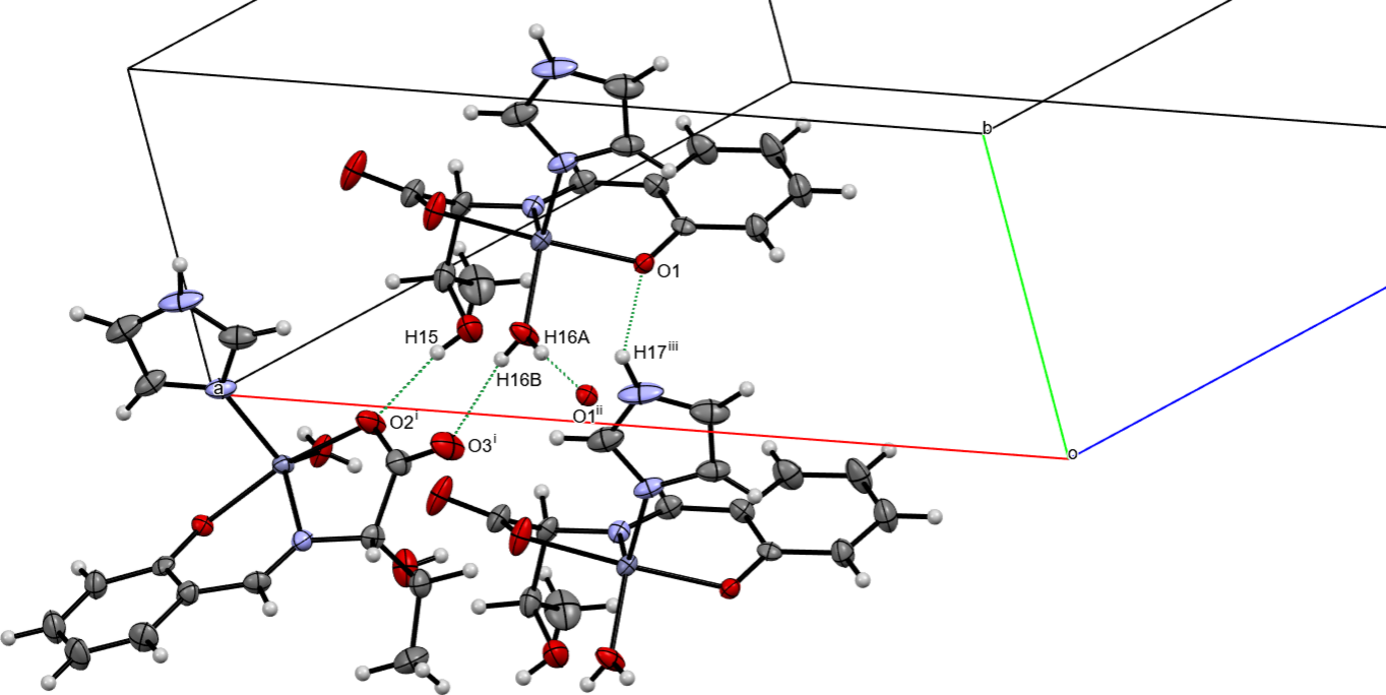
A view of the O—H⋯O and N—H⋯O hydrogen bonds, shown as dashed lines. [Symmetry codes: (i) −*x* + 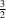, *y* − 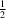, −*z*; (ii) −*x* + 1, *y*, −*z*; (iii) *x*, *y* + 1, *z*.]

Four inter­molecular hydrogen bonds are observed in the crystal (Fig. 2 ▸); two hydrogen bonds (O5—H16*A*⋯O1 and O5H—H16*B*⋯O3) lead to the formation of a chain structure along the *a*-axis direction. One hydrogen bond (O4—H15⋯O2) is formed along the *c*-axis direction (Table 1 ▸). In addition, an inter­molecular N3—H17⋯O1 inter­action is found (symmetry codes given in Table 1 ▸).

**Figure 3:**
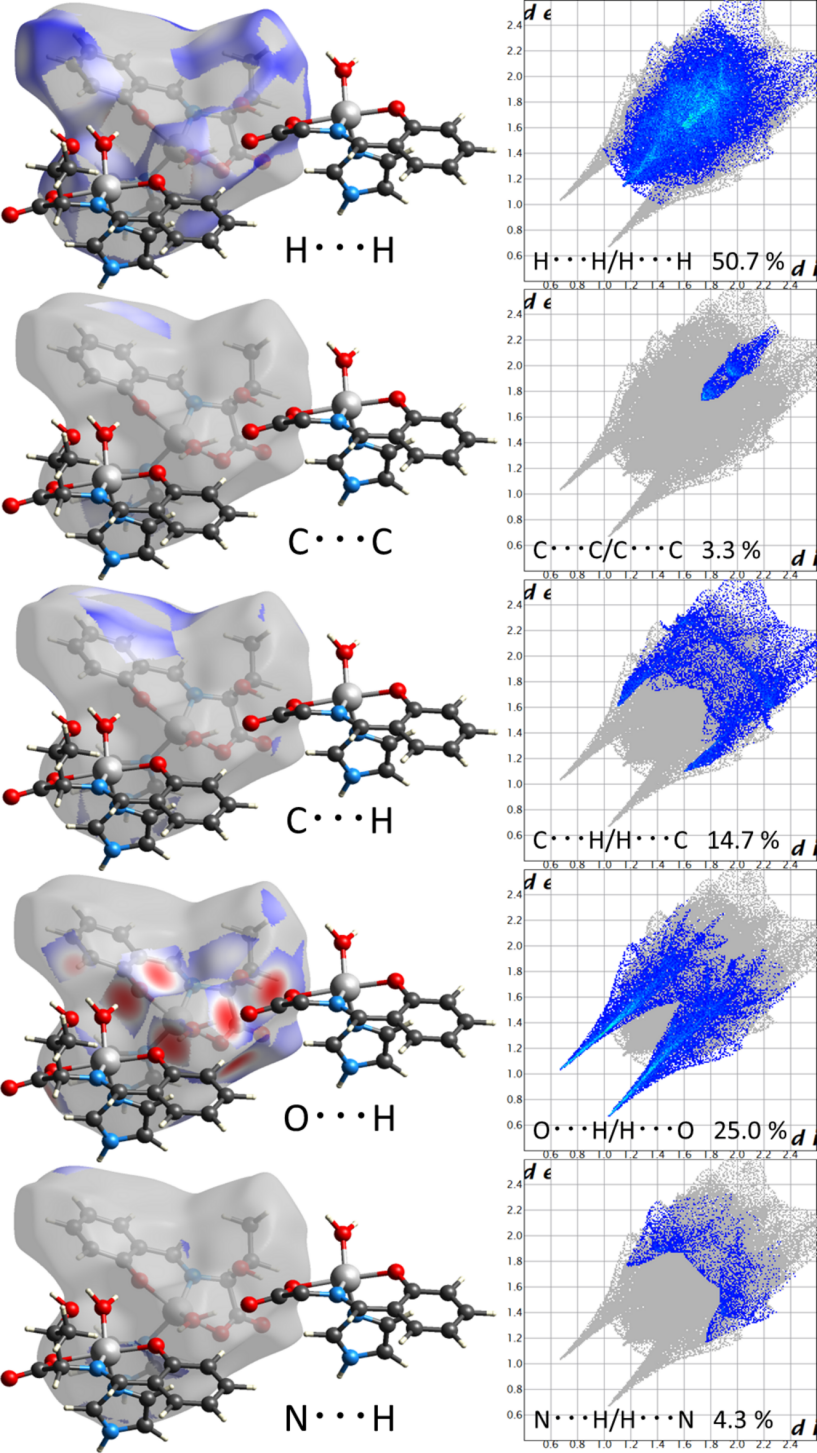
Hirshfeld surfaces mapped over *d*_norm_ and the two-dimensional fingerprint plots.

A Hirshfeld surface analysis (McKinnon *et al.*, 2007 ▸; Spackman & Jayatilaka, 2009 ▸) was performed to further investigate the inter­molecular inter­actions and contacts. The inter­molecular O—H⋯O hydrogen bonds are indicated by bright red spots appearing near O on the Hirshfeld surfaces mapped over *d*_norm_ and by two sharp spikes of almost the same length in the region 1.6 Å < (*d*_e_ + *d*_i_) < 2.0 Å in the 2D fingerprint plots (Fig. 3 ▸).

The contributions to the packing from H⋯H, C⋯C, C⋯H/H⋯C, N⋯H/H⋯N, and H⋯O/O⋯H contacts are 50.7, 3.3, 14.9, 4.3 and 25.0%, respectively. The structure is characterized by high proportion of H⋯H inter­actions, where H⋯H are van der Waals inter­actions. The high value of C⋯H/H⋯C is thought to arise from C—H⋯π inter­actions due to the presence of aromatic rings in the compound. The low value of C⋯C is the result of the low contribution of π–π stacking due to non-overlapping aromatic rings in the structure.

## 4. Database survey

A search in the Cambridge Structural Database (CSD, Version 5.43, update of November 2021; Groom *et al.*, 2016 ▸) for similar structures returned four relevant entries: aqua-[*N*-{[2-oxyphen­yl]methyl­idene}threoninato]-(methanol)copper(II) (YUYFUW; Katsuumi *et al.*, 2020 ▸), oxonium bis­{2-[(tetra­hydro­furan-2-ylmeth­yl)carbonoimido­yl]phenolato}zinc(II) perchlorate (KOVRAQ; Mandal *et al.*, 2014 ▸), (3-(4-hy­droxy­phen­yl)-2-{[(2-oxidophen­yl)methyl­idene]amino}­propano­ato)(1*H*-imidazole)­copper(II) (GIQWUC; Suzuki *et al.*, 2023 ▸), mono/bis­(aqua-κ*O*)[*N*-(2-oxido­benz­ylidene)valinato-κ^3^*O*,*N*,*O*′]copper(II) (VEXZIL; Akiyama *et al.*, 2023 ▸).

## 5. Synthesis and crystallization

l-threonine (11.912 mg, 0.10 mmol) was reacted with salicyl­aldehyde (12.212 mg, 0.10 mmol) in methanol (5 mL) and water (2 mL), and the resulting mixture was stirred at 313 K for 1 h to afford a yellow solution. To this solution, zinc(II) acetate dihydrate (21.951 mg, 0.100 mmol) was added and it was stirred at 313 K for 1 h. Then imidazole (6.808 mg, 0.10 mmol) was added, yielding a pale-yellow solution. For crystallization, the solution was placed in air at 300 K for several days, and the title complex was obtained as pale yellow columnar-shaped single crystals suitable for single-crystal X-ray diffraction structure analysis. All reagents are commercially available, but l-threonine moiety may partially racemize during synthesis. IR (ATR): 1070 cm^−1^(*w*), 1284 cm^−1^(*m*), 1376 cm^−1^(*m*), 1473 cm^−1^(*m*), 1475 cm^−1^(*m*), 1548 cm^−1^(*w*, C=C double bond), 1622 cm^−1^(*s*, C=O double bond), 1634 cm^−1^(*s*, C=N double bond), 3251 cm^−1^(*br*, O—H). UV-vis (H_2_O): 270 nm (*ɛ* = 38000 L mol^−1^ cm^−1^, π–π*); 359 nm (*ɛ* = 18000 L mol^−1^ cm^−1^, *n*–π*).

## 6. Refinement

Crystal data, data collection and structure refinement details are summarized in Table 2 ▸. All C-bound H atoms were placed in geometrically calculated positions (C—H = 0.94–1.00 Å) and were constrained using a riding model with *U*_iso_(H) = 1.2*U*_eq_(C) for *R*_2_CH and *R*_3_CH H atoms and 1.5*U*_eq_(C) for the methyl H atoms. The N-bound H atom H17 was constrained using a riding model with *U*_iso_(H) = 1.2*U*_eq_(N), and the O-bound H atoms H15, H16*A*, H16*B* were located based on a difference-Fourier map and refined freely.

## Supplementary Material

Crystal structure: contains datablock(s) global, I. DOI: 10.1107/S2056989025002385/ox2014sup1.cif

Structure factors: contains datablock(s) I. DOI: 10.1107/S2056989025002385/ox2014Isup2.hkl

CCDC reference: 2431599

Additional supporting information:  crystallographic information; 3D view; checkCIF report

## Figures and Tables

**Table 1:** Hydrogen-bond geometry (Å, °)

*D*—H⋯*A*	*D*—H	H⋯*A*	*D*⋯*A*	*D*—H⋯*A*
O4—H15⋯O2^i^	0.79 (7)	1.89 (7)	2.678 (4)	170 (8)
O5—H16*A*⋯O1^ii^	0.79 (7)	1.91 (7)	2.708 (4)	178 (6)
O5—H16*B*⋯O3^i^	0.72 (8)	2.02 (8)	2.724 (4)	165 (7)
N3—H17⋯O1^iii^	0.88	2.06	2.830 (6)	145

Symmetry codes: (i) 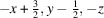; (ii) 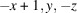; (iii) 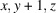.

**Table 2:** Experimental details

Crystal data	
Chemical formula	[Zn(C_11_H_11_NO_4_)(C_3_H_4_N_2_)(H_2_O)]
*M*_r_	372.67
Crystal system, space group	Monoclinic, *C*2
Temperature (K)	173
*a*, *b*, *c* (Å)	18.3835 (7), 7.7141 (3), 13.3800 (5)
β (°)	123.787 (1)
*V* (Å^3^)	1576.99 (11)
*Z*	4
Radiation type	Mo *K*α
μ (mm^−1^)	1.59
Crystal size (mm)	0.10 × 0.10 × 0.10

Data collection	
Diffractometer	Bruker-AXS D8 QUEST
Absorption correction	Multi-scan
*T*_min_, *T*_max_	0.64, 0.86
No. of measured, independent and observed [*I* > 2σ(*I*)] reflections	10846, 2752, 2701
*R*_int_	0.073
(sin θ/λ)_max_ (Å^−1^)	0.596

Refinement	
*R*[*F*^2^ > 2σ(*F*^2^)], *wR*(*F*^2^), *S*	0.032, 0.074, 1.07
No. of reflections	2752
No. of parameters	218
No. of restraints	1
H-atom treatment	H atoms treated by a mixture of independent and constrained refinement
Δρ_max_, Δρ_min_ (e Å^−3^)	0.35, −0.29
Absolute structure	Flack *x* determined using 1180 quotients [(*I*^+^)−(*I*^−^)]/[(*I*^+^)+(*I*^−^)] (Parsons *et al.*, 2013 ▸)
Absolute structure parameter	0.143 (11)

Computer programs: *APEX2* and *SAINT* (Bruker, 2019 ▸), *SHELXT2018/2* (Sheldrick, 2015*a* ▸), *SHELXL2019/1* (Sheldrick, 2015*b* ▸) and *ShelXle* (Hübschle *et al.*, 2011 ▸).

## supplementary crystallographic information

### Aqua(1H-imidazole-κN3)[N-(2-oxidobenzylidene)threonato-κ3O,N,O']zinc(II) Crystal data

**Table:**

[Zn(C_11_H_11_NO_4_)(C_3_H_4_N_2_)(H_2_O)]	*F*(000) = 768
*M**_r_* = 372.67	*D*_x_ = 1.570 Mg m^−^^3^
Monoclinic, *C*2	Mo *K*α radiation, λ = 0.71073 Å
*a* = 18.3835 (7) Å	Cell parameters from 6515 reflections
*b* = 7.7141 (3) Å	θ = 3.0–25.0°
*c* = 13.3800 (5) Å	µ = 1.59 mm^−^^1^
β = 123.787 (1)°	*T* = 173 K
*V* = 1576.99 (11) Å^3^	Prism, yellow
*Z* = 4	0.10 × 0.10 × 0.10 mm

### Aqua(1H-imidazole-κN3)[N-(2-oxidobenzylidene)threonato-κ3O,N,O']zinc(II) Data collection

**Table:**

Bruker-AXS D8 QUEST diffractometer	2701 reflections with *I* > 2σ(*I*)
Detector resolution: 7.3910 pixels mm^-1^	*R*_int_ = 0.073
profile data from θ/2θ scans	θ_max_ = 25.1°, θ_min_ = 3.0°
Absorption correction: multi-scan	*h* = −21→21
*T*_min_ = 0.64, *T*_max_ = 0.86	*k* = −9→9
10846 measured reflections	*l* = −15→15
2752 independent reflections	

### Aqua(1H-imidazole-κN3)[N-(2-oxidobenzylidene)threonato-κ3O,N,O']zinc(II) Refinement

**Table:**

Refinement on *F*^2^	Hydrogen site location: mixed
Least-squares matrix: full	H atoms treated by a mixture of independent and constrained refinement
*R*[*F*^2^ > 2σ(*F*^2^)] = 0.032	*w* = 1/[σ^2^(*F*_o_^2^) + (0.0364*P*)^2^] where *P* = (*F*_o_^2^ + 2*F*_c_^2^)/3
*wR*(*F*^2^) = 0.074	(Δ/σ)_max_ < 0.001
*S* = 1.07	Δρ_max_ = 0.35 e Å^−^^3^
2752 reflections	Δρ_min_ = −0.29 e Å^−^^3^
218 parameters	Absolute structure: Flack *x* determined using 1180 quotients [(*I*^+^)-(*I*^-^)]/[(*I*^+^)+(*I*^-^)] (Parsons *et al.*, 2013)
1 restraint	Absolute structure parameter: 0.143 (11)

### Aqua(1H-imidazole-κN3)[N-(2-oxidobenzylidene)threonato-κ3O,N,O']zinc(II) Special details

**Table:**

Geometry. All esds (except the esd in the dihedral angle between two l.s. planes) are estimated using the full covariance matrix. The cell esds are taken into account individually in the estimation of esds in distances, angles and torsion angles; correlations between esds in cell parameters are only used when they are defined by crystal symmetry. An approximate (isotropic) treatment of cell esds is used for estimating esds involving l.s. planes.

### Aqua(1H-imidazole-κN3)[N-(2-oxidobenzylidene)threonato-κ3O,N,O']zinc(II) Fractional atomic coordinates and isotropic or equivalent isotropic displacement parameters (Å^2^)

**Table:**

	*x*	*y*	*z*	*U*_iso_*/*U*_eq_	
Zn01	0.65438 (2)	0.42759 (7)	0.11898 (3)	0.01670 (17)	
O1	0.59603 (16)	0.2984 (3)	0.1916 (2)	0.0174 (5)	
O2	0.74153 (18)	0.5370 (4)	0.0799 (3)	0.0323 (7)	
O3	0.8834 (2)	0.5634 (5)	0.1533 (3)	0.0391 (8)	
O4	0.7676 (2)	0.1327 (5)	0.1191 (3)	0.0372 (8)	
O5	0.5804 (2)	0.3045 (4)	−0.0380 (3)	0.0276 (7)	
H16A	0.529 (5)	0.302 (8)	−0.085 (6)	0.041000*	
N1	0.7722 (2)	0.3625 (4)	0.2698 (3)	0.0196 (7)	
N2	0.6124 (2)	0.6638 (4)	0.1298 (3)	0.0241 (7)	
N3	0.6028 (3)	0.9438 (6)	0.1430 (3)	0.0420 (10)	
H17	0.611949	1.055498	0.141612	0.050000*	
H15	0.768 (5)	0.115 (10)	0.061 (6)	0.063000*	
C1	0.6293 (2)	0.2935 (5)	0.3089 (3)	0.0187 (7)	
C2	0.5734 (3)	0.2624 (6)	0.3481 (4)	0.0273 (9)	
H2	0.512602	0.246220	0.290352	0.033000*	
C3	0.6056 (3)	0.2549 (7)	0.4692 (4)	0.0365 (11)	
H3	0.566229	0.235803	0.492934	0.044000*	
C4	0.6946 (3)	0.2747 (7)	0.5573 (4)	0.0372 (11)	
H4	0.716190	0.269067	0.640331	0.045000*	
C5	0.7501 (3)	0.3026 (7)	0.5208 (4)	0.0339 (10)	
H5	0.810969	0.314747	0.580187	0.041000*	
C6	0.7204 (3)	0.3139 (5)	0.3988 (3)	0.0221 (8)	
C7	0.7866 (3)	0.3359 (6)	0.3735 (3)	0.0230 (8)	
H7	0.845966	0.330118	0.439697	0.028000*	
C8	0.8438 (2)	0.3703 (5)	0.2529 (3)	0.0225 (8)	
H8	0.899618	0.402041	0.330026	0.027000*	
C9	0.8220 (3)	0.5031 (6)	0.1559 (4)	0.0268 (9)	
C10	0.8523 (3)	0.1890 (6)	0.2094 (4)	0.0297 (9)	
H10	0.887625	0.201059	0.173851	0.036000*	
C11	0.8964 (4)	0.0585 (8)	0.3105 (6)	0.0507 (14)	
H11A	0.955393	0.099246	0.372280	0.076000*	
H11B	0.900354	−0.053288	0.279021	0.076000*	
H11C	0.862170	0.045004	0.345727	0.076000*	
C12	0.5546 (3)	0.6991 (6)	0.1608 (4)	0.0295 (9)	
H12	0.523720	0.613729	0.174295	0.035000*	
C13	0.5472 (3)	0.8718 (6)	0.1695 (4)	0.0381 (11)	
H13	0.511549	0.930343	0.189603	0.046000*	
C14	0.6408 (3)	0.8169 (7)	0.1196 (4)	0.0346 (10)	
H14	0.681965	0.833681	0.098842	0.041000*	
H16B	0.599 (5)	0.243 (10)	−0.059 (6)	0.052000*	

### Aqua(1H-imidazole-κN3)[N-(2-oxidobenzylidene)threonato-κ3O,N,O']zinc(II) Atomic displacement parameters (Å^2^)

**Table:**

	*U*^11^	*U*^22^	*U*^33^	*U*^12^	*U*^13^	*U*^23^
Zn01	0.0144 (2)	0.0149 (2)	0.0197 (2)	−0.00040 (18)	0.00884 (17)	0.00248 (17)
O1	0.0165 (12)	0.0172 (13)	0.0178 (11)	−0.0024 (11)	0.0091 (10)	−0.0005 (10)
O2	0.0171 (14)	0.0431 (19)	0.0350 (15)	0.0022 (13)	0.0133 (12)	0.0193 (14)
O3	0.0215 (15)	0.045 (2)	0.0486 (18)	−0.0046 (14)	0.0184 (13)	0.0176 (16)
O4	0.0358 (17)	0.045 (2)	0.0399 (17)	−0.0091 (15)	0.0269 (15)	−0.0134 (16)
O5	0.0164 (14)	0.0383 (19)	0.0231 (14)	0.0040 (14)	0.0080 (12)	−0.0115 (13)
N1	0.0190 (15)	0.0173 (14)	0.0238 (15)	0.0012 (13)	0.0127 (13)	0.0015 (12)
N2	0.0222 (16)	0.0146 (17)	0.0257 (16)	−0.0017 (13)	0.0073 (13)	0.0010 (13)
N3	0.048 (2)	0.0124 (18)	0.0373 (18)	0.0022 (19)	0.0066 (16)	−0.0011 (18)
C1	0.0222 (18)	0.0121 (17)	0.0214 (17)	0.0030 (15)	0.0119 (15)	−0.0014 (14)
C2	0.025 (2)	0.034 (2)	0.029 (2)	0.0011 (18)	0.0186 (17)	−0.0006 (18)
C3	0.037 (2)	0.050 (3)	0.034 (2)	0.002 (2)	0.027 (2)	0.001 (2)
C4	0.038 (2)	0.057 (3)	0.0196 (19)	0.007 (2)	0.0182 (18)	0.002 (2)
C5	0.030 (2)	0.045 (3)	0.0198 (19)	0.004 (2)	0.0099 (17)	0.0006 (19)
C6	0.0226 (18)	0.024 (2)	0.0195 (17)	0.0026 (16)	0.0113 (15)	−0.0012 (15)
C7	0.0178 (17)	0.024 (2)	0.0192 (18)	0.0008 (15)	0.0053 (15)	0.0020 (15)
C8	0.0116 (16)	0.027 (2)	0.0259 (18)	−0.0022 (15)	0.0085 (15)	0.0012 (15)
C9	0.022 (2)	0.026 (2)	0.034 (2)	−0.0045 (18)	0.0170 (18)	0.0028 (18)
C10	0.025 (2)	0.033 (2)	0.039 (2)	0.0010 (18)	0.0230 (19)	0.0036 (19)
C11	0.062 (3)	0.036 (3)	0.066 (3)	0.023 (3)	0.042 (3)	0.018 (3)
C12	0.024 (2)	0.021 (2)	0.033 (2)	0.0022 (17)	0.0095 (18)	−0.0052 (17)
C13	0.035 (2)	0.026 (2)	0.039 (2)	0.0076 (18)	0.0116 (19)	−0.0052 (18)
C14	0.036 (2)	0.023 (3)	0.030 (2)	−0.003 (2)	0.0088 (19)	0.0024 (17)

### Aqua(1H-imidazole-κN3)[N-(2-oxidobenzylidene)threonato-κ3O,N,O']zinc(II) Geometric parameters (Å, º)

**Table:**

Zn01—O5	1.996 (3)	N3—C14	1.337 (7)
Zn01—N2	2.015 (3)	N3—C13	1.371 (7)
Zn01—N1	2.038 (3)	C1—C2	1.410 (6)
Zn01—O1	2.060 (2)	C1—C6	1.427 (5)
Zn01—O2	2.117 (3)	C2—C3	1.383 (6)
O1—C1	1.331 (4)	C3—C4	1.394 (7)
O2—C9	1.272 (5)	C4—C5	1.371 (7)
O3—C9	1.238 (5)	C5—C6	1.407 (6)
O4—C10	1.408 (6)	C6—C7	1.443 (6)
N1—C7	1.276 (5)	C8—C9	1.522 (6)
N1—C8	1.456 (5)	C8—C10	1.556 (6)
N2—C14	1.327 (6)	C10—C11	1.509 (7)
N2—C12	1.366 (6)	C12—C13	1.351 (7)
			
O5—Zn01—N2	116.22 (13)	C2—C1—C6	117.4 (3)
O5—Zn01—N1	130.65 (13)	C3—C2—C1	121.2 (4)
N2—Zn01—N1	112.96 (13)	C2—C3—C4	121.5 (4)
O5—Zn01—O1	92.14 (12)	C5—C4—C3	118.1 (4)
N2—Zn01—O1	94.77 (12)	C4—C5—C6	122.5 (4)
N1—Zn01—O1	87.64 (11)	C5—C6—C1	119.2 (4)
O5—Zn01—O2	95.52 (13)	C5—C6—C7	116.4 (4)
N2—Zn01—O2	91.69 (14)	C1—C6—C7	124.2 (3)
N1—Zn01—O2	79.03 (12)	N1—C7—C6	125.5 (4)
O1—Zn01—O2	166.60 (11)	N1—C8—C9	109.2 (3)
C1—O1—Zn01	123.4 (2)	N1—C8—C10	108.0 (3)
C9—O2—Zn01	115.1 (3)	C9—C8—C10	108.7 (3)
C7—N1—C8	121.1 (3)	O3—C9—O2	124.9 (4)
C7—N1—Zn01	125.5 (3)	O3—C9—C8	117.8 (4)
C8—N1—Zn01	113.0 (2)	O2—C9—C8	117.2 (3)
C14—N2—C12	105.7 (4)	O4—C10—C11	110.5 (4)
C14—N2—Zn01	127.5 (3)	O4—C10—C8	107.7 (3)
C12—N2—Zn01	126.5 (3)	C11—C10—C8	112.3 (4)
C14—N3—C13	108.9 (5)	C13—C12—N2	110.8 (5)
O1—C1—C2	119.4 (3)	C12—C13—N3	104.6 (5)
O1—C1—C6	123.1 (3)	N2—C14—N3	110.0 (4)

### Aqua(1H-imidazole-κN3)[N-(2-oxidobenzylidene)threonato-κ3O,N,O']zinc(II) Hydrogen-bond geometry (Å, º)

**Table:**

*D*—H···*A*	*D*—H	H···*A*	*D*···*A*	*D*—H···*A*
O4—H15···O2^i^	0.79 (7)	1.89 (7)	2.678 (4)	170 (8)
O5—H16*A*···O1^ii^	0.79 (7)	1.91 (7)	2.708 (4)	178 (6)
O5—H16*B*···O3^i^	0.72 (8)	2.02 (8)	2.724 (4)	165 (7)
N3—H17···O1^iii^	0.88	2.06	2.830 (6)	145
C14—H14···O2	0.95	2.61	3.077 (6)	111
C13—H13···O3^iv^	0.95	2.36	3.253 (6)	156
C2—H2···O3^v^	0.95	2.48	3.351 (5)	152

Symmetry codes: (i) −*x*+3/2, *y*−1/2, −*z*; (ii) −*x*+1, *y*, −*z*; (iii) *x*, *y*+1, *z*; (iv) *x*−1/2, *y*+1/2, *z*; (v) *x*−1/2, *y*−1/2, *z*.
